# Case Report: Disorder of Sexual Development in a Chinese Crested Dog With XX/XY Leukocyte Chimerism and Mixed Cell Testicular Tumors

**DOI:** 10.3389/fvets.2022.937991

**Published:** 2022-07-08

**Authors:** Rebecca Schwartz, Nicole J. Sugai, Kristin Eden, Caitlin Castaneda, Matthew Jevit, Terje Raudsepp, Julie T. Cecere

**Affiliations:** ^1^Virginia-Maryland Regional College of Veterinary Medicine, Blacksburg, VA, United States; ^2^Department of Veterinary Clinical Sciences, Virginia-Maryland Regional College of Veterinary Medicine, Blacksburg, VA, United States; ^3^Virginia Tech Animal Laboratory Services, Virginia-Maryland Regional College of Veterinary Medicine, Blacksburg, VA, United States; ^4^Department of Basic Science Education, Virginia Tech Carilion School of Medicine, Roanoke, VA, United States; ^5^Department of Veterinary Integrative Biosciences, Molecular Cytogenetics Laboratory, College of Veterinary Medicine and Biomedical Sciences, Texas A&M University, College Station, TX, United States

**Keywords:** chimerism, disorder of sexual development (DSD), Sertoli cell tumor, Leydig cell tumor, canine

## Abstract

A 10-year-old intact female Chinese Crested dog was presented for evaluation and further diagnostics due to persistent symptoms of vulvar swelling, vaginal discharge, and an 8-year history of acyclicity. At presentation, generalized hyperpigmentation and truncal alopecia were identified, with no aberrations of the female phenotype. Vaginal cytology confirmed the influence of estrogen at multiple veterinary visits, and hormonal screening of progesterone and anti-Mullerian hormone indicated gonadal presence. Based on findings from abdominal laparotomy and gonadectomy, the tissue was submitted for histopathology. Histopathologic evaluation identified the gonads to be abnormal testes containing multiple Sertoli and interstitial (Leydig) cell tumors. The histopathologic diagnosis of testes and concurrent normal external female phenotype in the patient lead to a diagnosis of a disorder of sexual development (DSD). Karyotype evaluation by conventional and molecular analysis revealed a two cell line chimeric pattern of 78,XX (80%) and 78,XY (20%) among blood leukocytes, as well as a positive PCR test for the Y-linked *SRY* gene. Cytogenetic analysis of skin fibroblasts revealed the presence of 78,XX cells exclusively, and PCR tests for the Y-linked *SRY* gene were negative in the hair and skin samples. These results are consistent with an XX/XY blood chimerism. This is one of the few case reports of a canine with the diagnosis of leukocyte chimerism with normal female phenotypic external genitalia. This case illustrates a distinct presentation for hormonally active Sertoli cell tumorigenesis and demonstrates surgery as a curative treatment option for clinically affected patients.

## Background

The sexual determination of an individual animal begins at syngamy and continues through embryogenesis and organogenesis. The first step is establishment of the chromosomal sex and begins the differentiation process. Sexual differentiation continues in sequential order to gonadal and phenotypical differentiation throughout gestation as the fetus responds to the hormonal or molecular signaling for normal development (1, 2). In canines, the initiation of gonadal sexual differentiation begins around day 35–40 in gestation for distinguishing cell lines for male or female morphology (1). The gonadal ridge develops around day 30–35 in gestation and this undifferentiated ridge will migrate and transform according to specific inputs for female vs. male morphology. The gonadal portion of sexual differentiation directs primordial germ cell and embryologic tissue toward determining gonadal sex as testes or ovaries (1, 3). The sexual determination process concludes with the development of the concomitant genital for the external and internal phenotypic sex. Deviations in this cascade of sexual determination and differentiation, which involves complicated and precise interactions between genes, proteins, hormones, and receptors, result in various disorders of sexual development (DSDs). These disorders can occur at any of the three levels (2–4). Animals diagnosed with DSDs can present for a wide variety of reasons but typically possess some degree of abnormal external genitalia, or phenotypic sex (2–4). This case report describes the reproductive investigation and diagnosis of a dog presenting with normal female external genitalia and exclusively male gonadal tissue.

## Case Presentation

A 10-year-old female intact powderpuff Chinese Crested was presented to the Theriogenology service at the Virginia-Maryland College of Veterinary Medicine Teaching Hospital (VMCVM) for a referral evaluation. The patient had a history of intermittent to persistent symptoms of vulvar swelling, opaque mucoid vaginal discharge, and increased attention from an intact male dog in the home beginning about 8 months prior. These clinical signs were intermittent for a total of 15 months but peaked on average every 6 months. Previously, the owner reported never noticing estrous cyclicity in the patient. The patient had been with her owner for about 8 years, with no documented ovariohysterectomy procedure performed. The only surgical history noted were hemilaminectomies (cervical spine) twice, and abdominal exploratory surgery with removal of a foreign body. At the time of the abdominal surgery no reproductive structures within the abdomen were noted by the surgeon nor scarring of the linea alba. There was also a progressive history of hyperpigmentation and truncal alopecia along the ventrum and hind limbs over the last 12 months, as reported by the owner.

**Figure 1:**
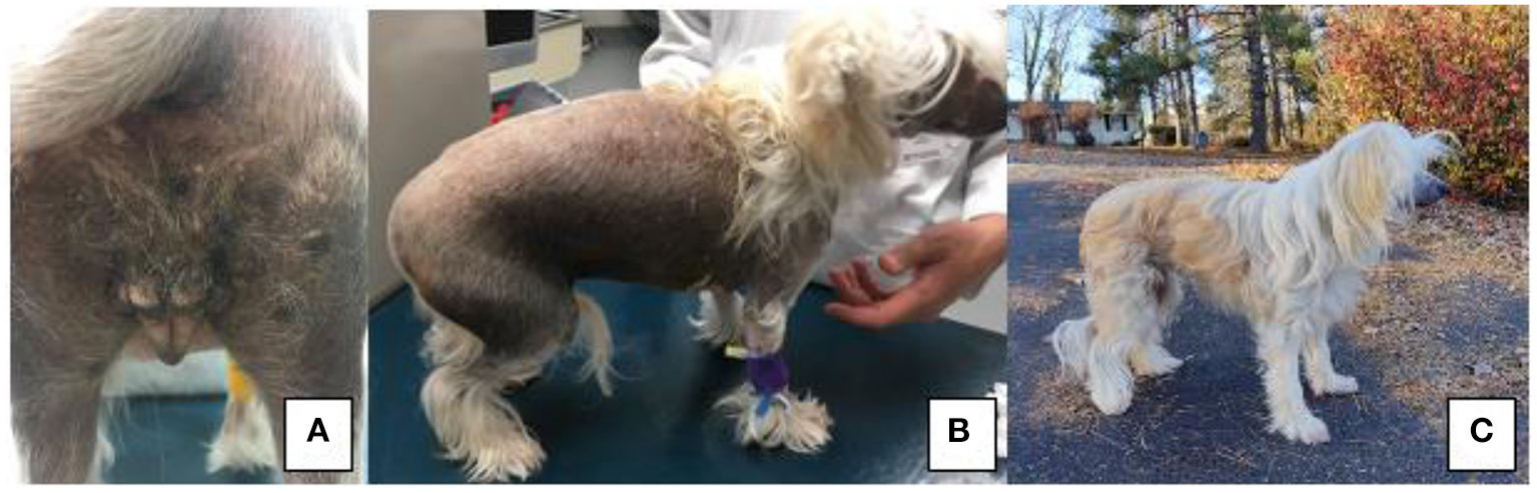
**(A)** Perineal region and vulva of patient on presentation to VMCVM; **(B)** Noted bilateral, non-pruritic alopecia on presentation of patient to VMCVM, and **(C)** Patient 6 months after recovering from surgery—noted significant hair regrowth.

On presentation, the patient had vulvar swelling (Figure 1A) as well as generalized alopecia with hyperpigmentation (Figure 1B). Initial diagnostics included a complete blood count and serum chemistry, vaginal cytology, cystocentesis for urinalysis and culture, and abdominal ultrasound. The patient's complete blood count and urinalysis resulted in no clinically significant findings, and urine culture yielded no growth at 24 h. Vaginal cytology consisted of superficial and intermediate epithelial cells with some inflammatory cells seen, which indicated estrogen influence. Abdominal ultrasound revealed a bladder with normal echogenicity and structure. Kidneys were bilaterally dilated. There was a 1.5 cm ovoid well-defined echogenic focus with multiple round to linear anechoic foci at the cranial aspect of the presumed uterine stump. A thin projection extended from the right aspect of this structure cranially, however this could only be traced for a short distance. These perceived uterine stump changes were interpreted to be due to remodeling secondary to persistent hormonal stimulation given the clinical history or potential neoplasia. No ovarian tissue was identified at this time. Due to insufficient serum samples, Anti-Mullerian Hormone (AMH) testing did not occur at this visit. Following up with their primary care veterinarian, this serum sample was submitted and was reported as a “very strong positive” for AMH based on enzyme-linked immunosorbent assay (ELISA), confirming gonadal presence.

On presentation for an abdominal exploratory surgery, a grade III out of VI heart murmur heard on both sides with equal intensity was noted which was not present on previous visits. An echocardiogram was performed, and the patient was diagnosed with myxomatous valvular degeneration (MMVD)-ACVIM stage B1 and cleared for surgery. Otherwise, her clinical signs were static from initial presentation to VMCVM.

**Figure 2:**
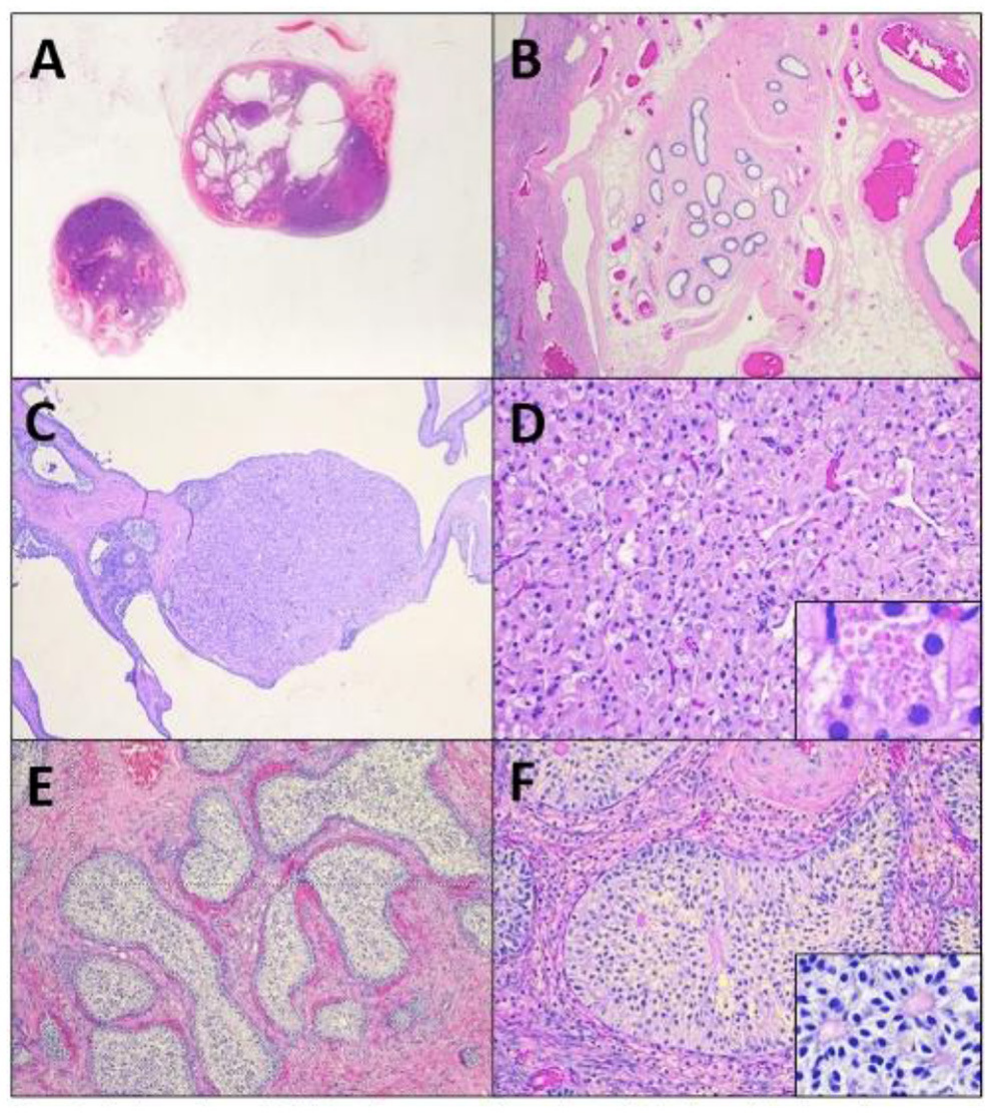
A subgross image **(A)** of the tissues reveals irregular testicular tissue with alternating areas of solid masses and cystic change, focally bordered by epididymal and vascular structures **(B)**. Within the tissue are multifocal Leydig cell tumors **(C)** characterized by nests of polygonal cells with granular to vacuolated eosinophilic cytoplasm **(D)**, that occasionally contain eosinophilic granules (**D**, insert). Remaining tubules are expanded by massively proliferative Sertoli cells forming intratubular neoplasi **(E)**, characterized by haphazard piles of elongated cells **(F)** that occasionally palisade around a central eosinophilic matrix (**F**, insert).

The following day, the patient was presented for an abdominal explore surgery. Two presumed gonadal structures were located near the level of the urinary bladder (Figure 2A). These tissues were ligated and excised. Their tubular tracts were followed caudally to the level of the pelvic inlet and ligated. The tissues were submitted for histopathologic evaluation. The incisions were closed in a routine three-layer manner. The patient was recovered in the hospital and sent home the same day with post-operative Meloxicam and standard recommendations for routine post-operative care.

Histopathologic evaluation revealed extremely irregular testicular tissue with distinct features (Figures 2B–F). The testicular tissue was composed of seminiferous tubules that were separated by connective tissue and interstitial cells with areas of dilated epididymal structures at the periphery. The seminiferous tubules contained no evidence of spermatogenesis or spermatogenic precursor cells but were instead lined by hyperplastic Sertoli cells that sometimes obscured the lumen. Multifocally, cells proliferated to such an extent that they formed multiple large, distinct intratubular neoplasms composed of elongate Sertoli cells arranged in concentric, haphazard piles with moderate amounts of eosinophilic cytoplasm, elongated nuclei with finely stippled chromatin, and indistinct nucleoli. Anisocytosis and anisokaryosis were mild and the mitotic index was 1 in 10 to 40 × fields. Neoplastic cells often surrounded central areas of eosinophilic matrix reminiscent of Call-Exner bodies. Multifocally throughout the remaining tissue, areas of seminiferous tubules were effaced and replaced by a second neoplasm composed of polygonal cells (interstitial cells) arranged in cords and vague nests supported by a fine fibrovascular stroma (Figures 2E,F). Neoplastic cells had distinct cell borders, moderate amounts of eosinophilic granular or clear vacuolated cytoplasm with eosinophilic globules, and 1–2 variable distinct nucleoli. Anisocytosis and anisokaryosis were moderate and the mitotic rate was <1 in 10 to 40 × fields (Figure 2F). Sections additionally contained large vascular structures analogous to pampiniform plexus. All the tubular tract tissue was composed of adipose tissue and vasculature suggestive of spermatic cord. Based on these findings, the diagnosis of diffuse spermatogenic atrophy with Sertoli Cell hyperplasia, multifocal intratubular Sertoli cell neoplasia, multifocal interstitial (Leydig) cell tumor and presumed disorder of sexual differentiation were assigned.

**Figure 3:**
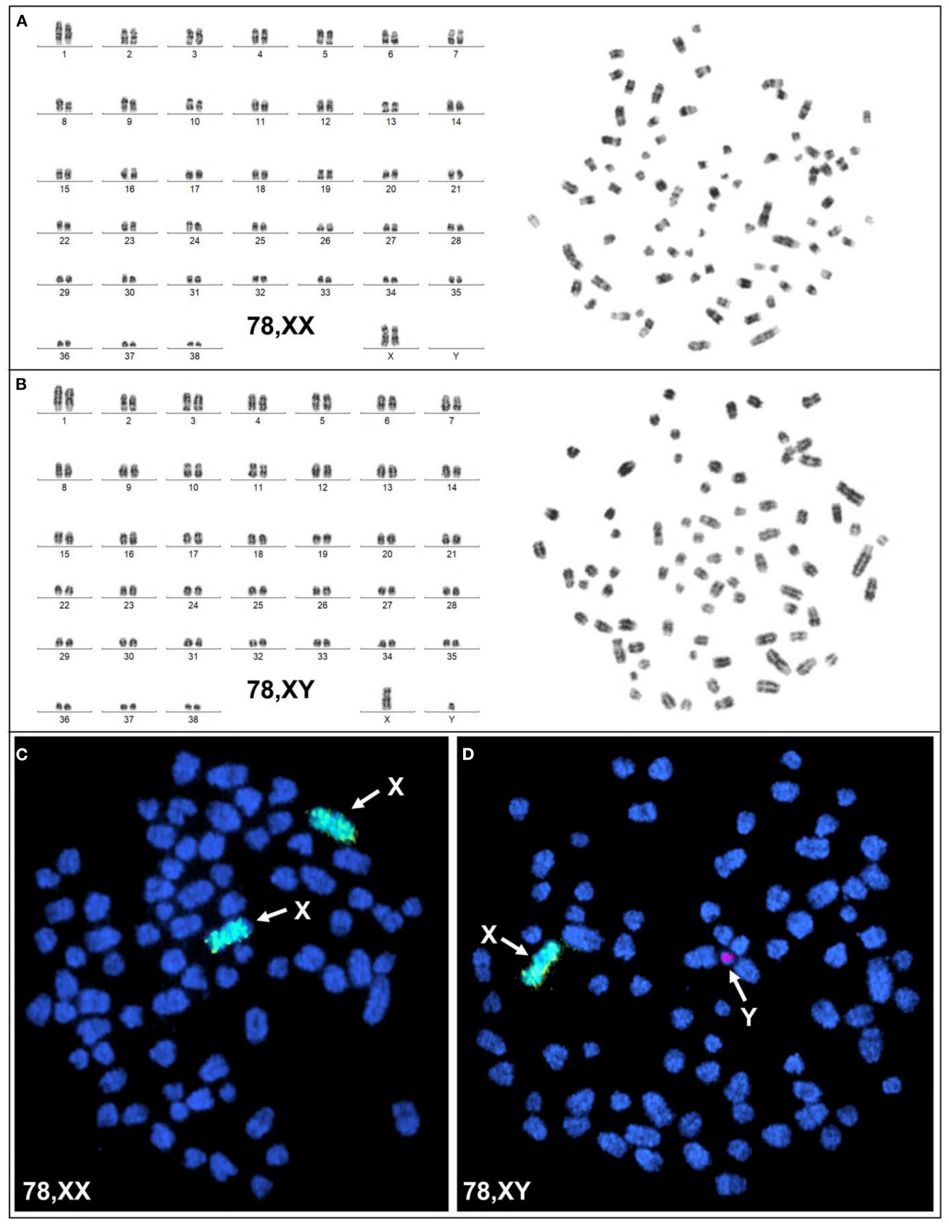
Cytogenetic and molecular cytogenetic analysis of blood leukocytes. **(A)** G-banded karyotype (left) and corresponding metaphase (right) of a 78,XX cell; **(B)** G-banded karyotype (left) and corresponding metaphase (right) of a 78,XY cell; **(C)** FISH with cat X chromosome painting probe (green; arrows) in a 78,XX cell, and **(D)** FISH with cat X chromosome painting probe (green; arrow) and dog Y-specific TSPY probe (red; arrow) in a 78,XY cell.

**Figure 4:**
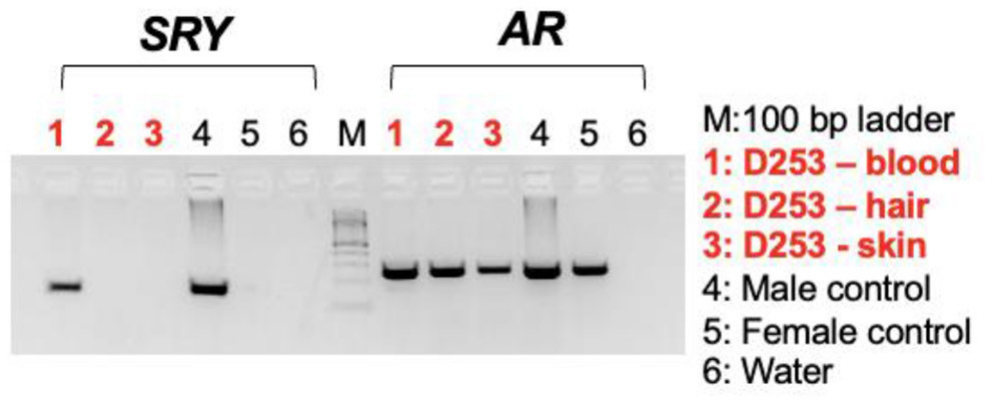
Agarose gel images showing PCR results with the sex determining region in Y, SRY (left panel) and X-linked androgen receptor gene, AR (control) on DNA templates from blood lymphocytes, hair, and skin fibroblasts together with male and female controls.

Karyotyping and molecular cytogenetic analysis of sex chromosomes by fluorescence *in situ* hybridization (FISH) was conducted from blood lymphocyte and primary skin fibroblast cultures as described elsewhere (Figure 3) (5). Primary skin fibroblasts were obtained from skin biopsies that were performed using local 2% lidocaine block and 5 mm punch biopsy instrument. Chromosomes were routinely stained with Giemsa which allowed counting the diploid number, identification of the sex chromosomes, and arrangement of autosomes into a karyotype according to size. Metaphases were also G-banded with trypsin (6) and arranged into karyotypes following the nomenclature proposed by Graphodatsky et al. (7). For sex chromosome analysis by FISH, we used a flow-sorted biotin-labeled X chromosome painting probe of a domestic cat (8) and a digoxigenin-labeled canine *TSPY*-specific probe (9). Genomic DNA was isolated with Blood and Tissue DNA kit (Qiagen) from blood, skin, and hair. The DNA was tested for the Y-linked *SRY* gene by PCR using the following primers: F 5'-ATGAACGCATTCTTGGTGTG-3' and R 5'-TGCATGGCCTGTAGTCTCTG-3'; product size 170 base-pairs (bp). An X-linked Androgen Receptor (*AR*) gene served as a positive PCR control with the following primers: F 5'- GCTACCTGGCTCTGGATGAG-3' and R 5'- AAGGAGTTGCATGGTTCCAG-3'; product size 266 bp (Figure 4). A total of 55 metaphase cells were analyzed from blood, with 7 cells karyotyped. The conventional and molecular cytogenetic analyses revealed a two cell-line pattern of 78,XX (80%) and 78,XY (20%) among the blood leukocytes, as well as blood DNA being positive for *SRY* by PCR. Cytogenetic analysis of 50 metaphase spreads from skin fibroblasts revealed the presence of 78,XX cells only. PCR tests for *SRY* were negative in the hair and skin samples. Thus, this patient is a blood chimera of 80% 78,XX and 20% 78,XY cell ratio in blood leukocytes with only 78,XX (female) cells in the skin and hair.

At time of publication, the patient is doing well clinically at home. Since recovering from gonadectomy, the patient's clinical symptoms have resolved. The patient's skin and coat changes reversed, and the patient has a normal powderpuff coat (Figure 1C). At this time, no signs of vulvar swelling, vaginal discharge or exercise intolerance are appreciated at home.

## Discussion

This case report describes the diagnosis of a dog with a disorder of sexual development occurring at the gonadal level with no obvious external signs of the disorder. This patient can be classified as a 78,XX/78,XY blood chimera based on the genetic difference between blood leukocytes which contained both genetically female (XX) and male (XY) cells from skin fibroblasts and hair follicles which comprised of genetically female (XX) cells only (10). Based on the patient's clinical signs and history, the development of the mixed cell gonadal neoplasia with functional Sertoli cell function for hyperestrogenism is the cause of the clinical signs.

In the present study, genetic evaluation of the patient was conducted by karyotyping, FISH, and PCR which were appropriate and sufficient approaches to determine the genetic sex of cells in peripheral blood, skin fibroblasts, and hair follicles as well as for evaluating the ratio of XX and XY cells in blood. In a few previous studies, similar cases have been studied by genotyping for X- and Y-chromosome specific short tandem repeats (STRs), also known as microsatellite markers (11). While STR genotyping efficiently detects the presence of a small number of male (XY) cells in a female (XX) background, it has limitations for detecting female cells in a male background and in cases of XX/XY chimerism or mosaicism, it is not able to determine the ratio of XX and XY cells.

Disorders of sexual development (DSD) may occur at any stage from syngamy through organogenesis (2–4). According to the new classification system, DSDs are classified based on where the problem occurs, either at the chromosomal, gonadal or phenotypic level to describe the disorder (12). For sexual differentiation, the chromosomal sex is typically determined at fertilization when the oocyte and sperm nucleus form the pronucleus of the resultant embryo. From this stage, the next step in sexual differentiation is not determined until the bipotential gonadal ridge is migrating toward the anus for the female or near the umbilicus for male (2, 13). For gonadal sex differentiation, this is an active process for both sexes across the species. For canines, the gonadal sex differentiation typically begins around day 30 in gestation with migration of the bipotential gonadal ridge (2, 10, 13).

For male differentiation, transcription of the sex determining region Y, the *SRY* gene, must be upregulated to activate a cascade of other genes (*SOX9*) that trigger differentiation toward testis (3). With the *SRY* gene expression on the genital ridge, this allows the once bipotential ridge to begin development for male genitalia (13). Testicular differentiation is noted at day 36 of gestation in the dogs and at this time will be aligned with regression of the paramesonephric duct (2, 13). As the testes develop, the Sertoli cells will differentiate from the sex cord cells. The development of Sertoli cells allows them to become hormonally active to produce Anti-Mullerian Hormone (AMH). The production of AMH will induce the regression of the Mullerian (paramesonephric) ducts by day 36–46 of gestation in a typical male fetus (1–3). Similar timing, the Leydig cells develop in the fetal gonad from interstitial mesenchymal cells and will begin producing testosterone. Testosterone is metabolized to the active form of 5-alpha-dihydrotestosterone (DHT) which influences the internal and external genitalia formation (3, 13). For a typical male fetus, the external genitalia formation is hormonally driven (10, 13). This is a difference from the female phenotypic aspect of sexual differentiation, in respect to the tubular tract development (10). For the female differentiation, the absence of *SRY* gene and upregulation of the beta-catenin pathway are crucial for gonadal differentiation. Without *SRY* influence, *SOX9* and *FGF9* are not active in testes gonadal differentiation. Also, the expression of the X-linked gene, *DAX1*, aids in suppression of *SF1*. High levels of *WNT4* can interfere with *SOX9* expression which aids ovarian development. The *RSPO1* gene is active and works synergistically with *WNT4* to upregulate and stabilize the beta-catenin pathway for ovarian differentiation. Another factor, FOXL2 can bind to *SOX9* to prevent its activation allowing ovarian structural development (2, 3). For females, the Mullerian (paramesonephric) duct will develop into the uterus and cervix if there is not enough stimulation from the AMH or androgens insufficiency (2, 10). This condition is due to failure of masculinization of the target organs to be able to develop appropriately into the corresponding male external genitalia (1, 2).

In the presented case, the disorder occurred at the gonadal level based on the karyotype consistent with XX/XY leukocyte chimerism. Leukocyte chimerism XX/XY, a form of DSD, is caused by the formation of anastomoses between the placentas of heterosexual fetuses, enabling the exchange of hematopoietic cells and molecules such as testosterone and protein products of the *SRY* gene expression between the fetuses that are involved in sex differentiation (11). It is suspected that the exchange of hemopoietic progenitor cells between fetuses can result in blood chimera by transcription products are able to modify gonadal differentiation during specific periods of organogenesis (11, 14). The presence of *SRY* gene expression can down-regulate the beta-catenin pathway and inhibit ovarian gonad differentiation (2, 3, 15). This exchange needs to occur prior to or at the time of sexual differentiation to allow the masculinization of the female reproductive tract to form discordance at the gonadal level (15). The frequency of occurrence of XX/XY blood chimerism differs between species based on the type of placenta, with higher incidence of anastomoses occurring in ruminants with cotyledonary organized placentas, and lower incidences occurring in carnivores with zonary organized placentas (11). This is due to the increased surface area for fetal-maternal vasculature interface. Compared to zonary placenta structure that is localized for fetal-maternal implantation for exchange (15, 16). Research in sheep noted potential for polygenic sire linked heritability or multifactorial influence for placental vasculature anastomosis formation as discrepancy in prevalence among species (14). While further research is needed to understand this potential association, it has been suggested that this may also apply to zonary placenta species like dogs and pigs, where a greater number of puppies or piglets in a litter may be associated with a higher incidence of anastomoses and resulting in freemartin puppies (11). A case report noted that in a rare case of dizygotic twin with monochorion, this aberration allowed sufficient vascular anastomosis to produce freemartin twins (15). Due to the rarity of this condition in canines, the mechanism for enabling vascular anastomosis is not well understood compared to bovine cases. Multiple hypotheses are noted in the literature for the condition in litter bearing species (11, 15, 16).

While aberrations in the external genitalia are commonly initial catalysts in the diagnosis of DSD in dogs, in freemartin dogs, this is less useful, as these patients have presented with a variety of external genitalia appearances ranging from almost normal female, normal male, and ambiguous (11). Examples of phenotypic aberrations include juvenile vulva conformation, hypoplastic tubular tract, and enlarged clitoris (11). While some canines can have minimal to no aberration to external genitalia and can present for primary anestrus (11). In bovine and porcine cases, the female twin or littermate has some aberration to external and internal genitalia. Case reports of varying degree of masculinization of tubular tract such as short, blind ended vagina, hypoplastic uterine horns, shortened vulva length or juvenile vulva conformation (16). Mature adults can have varying degree of masculinization to general appearance such as thickened neck, larger stature, like bull conformation (16). The male twin in some bovine cases can have some degree of feminization based on when the anastomosis occurred in gestation. This can lead to infertility or early onset sterility in some bull calves (16). This case demonstrates a patient with completely normal female external genitalia with XX/XY leukocyte chimerism. Therefore, this case is unique in that the major clinical signs for this patient were due to the functional gonadal neoplasia that developed from the cryptorchid testes with no other overt signs of sexual development abnormality (3).

The patient's clinical signs that lead to their specialty referral were the result of feminizing syndrome due to the patient's retained testes and resultant hormonally active Sertoli cell tumors. In retained testes, there is a documented increased risk of testicular neoplasms such as Sertoli cell tumors (16). Sertoli cell tumors and seminomas occur about 13 times more frequently in cryptorchid dogs compared to normal dogs (17, 18). For Sertoli cell tumors arising from abdominal testes, these tumors are functional in about 70% of cases (19). The clinical signs are often associated with feminizing paraneoplastic syndrome that includes bilateral symmetrical alopecia, hyperpigmentation, and attraction to other males (17). In severe cases, estrogen myelotoxicosis occurs due to steroid producing neoplastic Sertoli cells (17, 18, 20).

The pathophysiology of hyperestrogenism from functional Sertoli cells tumors is likely multifactorial stemming from excess production of estrogen or excess inhibin secretion. With the neoplastic Sertoli cells producing and secreting inhibin, this will down regulate the production and secretion of follicular stimulating hormone (FSH) and luteinizing hormone (LH) from the anterior pituitary (20). This downregulation will inhibit the testosterone production at the Leydig cells and likely alter the estrogen to testosterone ratio at a systemic level. This is due to the peripheral tissues being able to continue to produce and convert testosterone to estrogen or estradiol derivatives (18, 20). As for hyperestrogenism, this can occur from multiple production sites in the body at a local site or peripheral locations. For local production of estrogen from the neoplastic cells in the testes, the androgens will be converted to estrogen at an excessive level which can lead to the clinical signs (21). With this canine case, the potential for androgen insufficiency or receptor insensitivity could factor into the mechanism for this individual for a functional Sertoli cell tumor as well. The impressive degree of Sertoli cell hyperplasia and multifocal instances of progression to intratubular neoplasia are likely related to the elevated AMH and signs of hyperestrogenism seen in the patient.

Often in cases of testicular tumors in canines, there is a mixed cell population present. Such is the case for this patient who had multifocal interstitial (Leydig) cell tumors and intratubular Sertoli cell neoplasia present in both retained testes. Cryptorchidism is not a predisposing factor for interstitial (Leydig) cell neoplasm, which more likely was an incidental finding in this case, as it is not typically associated with the presenting clinical signs (20, 22). Interstitial (Leydig) cell tumors are typically not functional or will not cause signs of hyperandrogenism. Metastasis is rare for both Sertoli cell tumors and Interstitial (Leydig) cell tumors (18). Surgical removal of affected testes is therefore curative in the majority of cases (18, 20–23). If there was metastasis from Sertoli cell tumors, this is typically to the regional lymph nodes (19). In this case, because reactive or large lymph nodes were not noted on abdominal ultrasound and the clinical signs resolved after gonadectomy, further diagnostics to rule out possible metastasis was not pursued.

## Concluding Remarks

This report documents the diagnosis, treatment and long term follow up of a dog with a disorder of sexual differentiation with mixed cell testicular neoplasia. This case highlights the variation in presentation for a disorder of sexual development and how clinical signs can present in older patients due to comorbidities.

## Data Availability Statement

The original contributions presented in the study are included in the article/supplementary material, further inquiries can be directed to the corresponding author.

## Author Contributions

RS and NS contributed to writing the manuscript and literature review. KE assessed the gross and histopathologic findings. JC contributed to the critical revision of the manuscript, assisted with the surgery on the patient, and managed the clinical case. CC, MJ, and TR contributed to the cytogenetic and *SRY* analysis and results. All authors contributed to the final review. All authors contributed to the article and approved the submitted version.

## Conflict of Interest

The authors declare that the research was conducted in the absence of any commercial or financial relationships that could be construed as a potential conflict of interest.

## Publisher's Note

All claims expressed in this article are solely those of the authors and do not necessarily represent those of their affiliated organizations, or those of the publisher, the editors and the reviewers. Any product that may be evaluated in this article, or claim that may be made by its manufacturer, is not guaranteed or endorsed by the publisher.
